# Direct and indirect associations between the family physical activity environment and sports participation among 10–12 year-old European children: testing the EnRG framework in the ENERGY project

**DOI:** 10.1186/1479-5868-10-15

**Published:** 2013-02-03

**Authors:** Anna F Timperio, Maartje M van Stralen, Johannes Brug, Elling Bere, Mai JM Chinapaw, Ilse De Bourdeaudhuij, Nataša Jan, Lea Maes, Yannis Manios, Luis A Moreno, Jo Salmon, Saskia J te Velde

**Affiliations:** 1 Centre for Physical Activity and Nutrition Research (C-PAN), Deakin University, 221 Burwood Hwy, Burwood, VIC 3125, Australia; 2 EMGO Institute for Health and Care Research and the Department of Public and Occupational Health, VU University Medical Center, Van der Boechorstraat 7, Amsterdam 1081 BT, the Netherlands; 3 EMGO Institute for Health and Care Research and the Department of Biostatistics and Epidemiology, VU University Medical Center, Van der Boechorstraat 7, Amsterdam 1081 BT, the Netherlands; 4 Department of Public Health, Sport and Nutrition, University of Agder, Gimlemoen 25, Kristiansand, Norway; 5 Department of Movement and Sport Sciences, Ghent University, Watersportlaan 2, Ghent 9000, Belgium; 6 Slovenian Heart Foundation, Dunajska 65, Ljubljana 1000, Slovenia; 7 Department of Public Health, Ghent University, Watersportlaan 2, Ghent, B-9000, Belgium; 8 Department of Nutrition and Dietetics, Harokopio University, 70, El Venizelou Ave, Athens, Kallithea 176 71, Greece; 9 GENUD (Growth, Exercise, Nutrition and Development) Research Group. E.U. Ciencias de la Salud, Universidad de Zaragoza C/Domingo Miral s/n, Zaragoza 50009, Spain

**Keywords:** Sport, Physical activity, Children, Family, Home, Determinants, Mediation, Cognitions

## Abstract

**Background:**

Sport participation makes an important contribution to children’s overall physical activity. Understanding influences on sports participation is important and the family environment is considered key, however few studies have explored the mechanisms by which the family environment influences children’s sport participation. The purpose of this study was to examine whether attitude, perceived behavioural control, health belief and enjoyment mediate associations between the family environment and 10–12 year-old children’s sports participation.

**Methods:**

Children aged 10–12 years ( = 7234) and one of their parents (n = 6002) were recruited from 175 schools in seven European countries in 2010. Children self-reported their weekly duration of sports participation, physical activity equipment items at home and the four potential mediator variables. Parents responded to items on financial, logistic and emotional support, reinforcement, modelling and co-participation in physical activity. Cross-sectional single and multiple mediation analyses were performed for 4952 children with complete data using multi-level regression analyses.

**Results:**

Availability of equipment (OR = 1.16), financial (OR = 1.53), logistic (OR = 1.47) and emotional (OR = 1.51) support, and parental modelling (OR = 1.07) were positively associated with participation in ≥ 30mins/wk of sport. Attitude, beliefs, perceived behavioural control and enjoyment mediated and explained between 21-34% of these associations. Perceived behavioural control contributed the most to the mediated effect for each aspect of the family environment.

**Conclusions:**

Both direct (unmediated) and indirect (mediated) associations were found between most family environment variables and children’s sports participation. Thus, family-based physical activity interventions that focus on enhancing the family environment to support children’s sport participation are warranted.

## Background

Childhood physical activity is associated with multiple health benefits, including the promotion of a healthy weight, bone health, social development, cognitive function and self-esteem [1], as well as lower risk of developing future obesity, diabetes and cardiovascular disease risk factors [2,3]. It is recommended that youth accumulate 60 minutes of physical activity each day [4]. Sport is a common form of physical activity in youth and can make an important contribution to their overall physical activity and energy expenditure [5-7] and future physical activity as an adult [8], as well as develop motor skills and provide opportunities for social interaction [9].

The family is considered the most important setting for shaping children’s physical activity [10]. Parental physical activity through modelling of physical activity or sport [11-18] and co-participation with children [13], parental support through accompanying children to sports training and events, providing money and clothing for activity and encouraging physical activity [13-15,19,20] and the physical environment within the home [21] may be particularly important. The Environmental Research framework for weight Gain prevention (EnRG) [22] suggests that the family environment may have direct and indirect effects on energy-balance behaviours such as physical activity. This dual-process conceptual framework proposes that direct pathways between the family environment and behaviours may be the result of a spontaneous, automatic response to an environmental cue within the family (automaticity), while indirect pathways may be mediated by individual ‘cognitive’ determinants or thought processes. Cognitions are among the most proximal modifiable influences on behaviour. The EnRG framework suggests cognitive factors predominantly from the Theory of Planned Behaviour (TPB), comprised of factors such as attitudes, subjective norms and perceived behavioural control, may mediate environment-behaviour associations [23]. Other theories also contribute cognitive constructs, such as enjoyment, that have been associated with children’s physical activity [24,25]. These psychological constructs provide a positive psychological state for engaging in health-related behaviour. Perceived behavioural control, like self-efficacy, refers to one’s belief that he/she is capable of performing a given behaviour [23], which may be developed through vicarious learning and persuasion [26]. This perception, in turn, may improve motivation and help individuals to initiate and maintain behaviour, and determine how much effort the person will make [23,26]. Understanding mechanisms by which the family environment is associated with children’s physical activity is important for furthering theoretical frameworks and developing effective interventions.

Few studies have examined how children’s personal cognitions mediate associations between the family environment and physical activity. In preschoolers, direct and indirect effects via child enjoyment of physical activity have been reported between family support and objectively measured physical activity [27]. Both direct and indirect associations via self-efficacy have been found for family support for physical activity [28] and family social influence (modelling and encouragement) [29] among adolescent girls and youth whose physical activity declined over four months, respectively. Motl et al. [30] also found evidence of an indirect association between access to physical activity equipment in the home and physical activity among adolescent girls operating via self-efficacy. In a more comprehensive study, van der Horst et al. [21] found that associations between equipment at home, family physical activity rules and parental sports participation and sports participation among adolescents were partly mediated by attitude and intention, with direct effects also noted for equipment at home and parental sports participation. Mediation via parental subjective norm or perceived behavioural control was not evident.

There is a dearth of research exploring cognitive pathways through which the family environment influences physical activity among children. Most research examines only one cognitive mediator and focuses predominantly on adolescents. Late childhood is an important age group as, while beginning to develop some independence, children are not autonomous and declines in physical activity tend to occur during the transition to adolescence and beyond [13,31]. This paper aims to identify direct and indirect (mediated) associations between aspects of the family environment and 10–12 year-old children’s weekly participation in sport. Specifically, it aims to determine whether a range of cognitive factors (attitude, perceived behavioural control, health beliefs and enjoyment) mediate associations between the family environment and sports participation.

## Methods

Data were drawn from the cross-European school-based survey component of the “EuropeaN Energy balance Research to prevent excessive weight Gain among Youth” (ENERGY) project [32,33]. The design and methodology of the survey component has been previously described [33] and only brief details are presented here. The survey was conducted in schools in seven European countries: Belgium (Flanders), Greece, Hungary, the Netherlands, Norway (southern regions), Slovenia and Spain (Aragón). Data collection involved child surveys completed during class-time, anthropometrics, and parent surveys completed unsupervised at home. Ethical approval was obtained in each country from relevant ethical committees and ministries: Belgium: Medical Ethics Committee of the University Hospital Ghent; Greece: Bioethics Committee of Harokopio University; Hungary: Scientific and Ethics Committee of Health Sciences Council; Netherlands: Medical Ethics Committee of the VU University medical center; Norway: National Committees for Research Ethics in Norway; Slovenia: National Medical Ethics Committee of the Republic of Slovenia; Spain: Clinical Research Ethics Committee of the Government of Aragón.

### Sample selection

A random, multi-staged procedure stratified by degree of urbanicity was used to sample schools in each country. Response rates among approached schools ranged from 5% in the Netherlands to 100% in Slovenia. In total, 175 schools participated with the number of schools per country ranging from 15 in Slovenia to 37 in Greece. Following school recruitment, parents of eligible children received a letter explaining the study and inviting participation. Written consent was required for their own and their child’s participation in the study in all countries except the Netherlands (where passive informed consent was allowed) [33]. The researchers did not specify which parent should take part; this decision was taken by parents. The response rates ranged between 33% in Hungary to 98% in Slovenia (mean response rate 94%), and among parents from 41% in Norway to 86% in Slovenia (mean response rate 79%). In total, questionnaires were completed by 7234 children (ranging between 926 and 1178 per country) and 6002 parents (ranging between 404 and 1028 per country) [33]. Recruitment and data collection occurred between March and July 2010.

### Measures

#### Sociodemographics

Children reported their sex, month and year of birth, the language most often spoken at home, and adults and siblings they live with. Parents reported the number of years of education they completed and their marital status.

#### Sports participation

Children were asked to nominate their favourite two sports and for each were asked how many hours in total they did that sport [33]. Ten response options ranging from 30 minutes/week (0.5) to 5 hours (5) a week or more, increasing in 30 minute increments, were presented, along with the option of no participation (0). Responses to each sport were summed. Test-retest reliability over a one-week period in a separate sample of 730 children indicated good to high agreement (ICC ≥ 0.74). Comparison with responses in a cognitive interview regarding behaviour over the course of a normal day indicated good construct validity in a further sample of 96 children (ICC = 0.61) [34]. Sports participation was dichotomised to distinguish between those who do and do not participate in sport (no participation; ≥ 30 min/wk).

#### Family environment

Seven aspects of the family environment were examined. Children were asked whether they have the following eight equipment items at home that they can use for physical activity/sport: bike; tennis and/or badminton racket; ball (basketball, volleyball, football, etc.); sporting shoes; skipping rope; skates; skis; skate board (85–91% agreement across items). These items were summed to compute an equipment score (range 0–8). Parents reported remaining aspects of the family environment. Parents were asked if they pay for their child to take part in physical activity/sports (financial support), bring their child to physical activity/sport sessions (logistic support); encourage their child to take part (emotional support); and praise their child if (s)he takes part (reinforcement).[33] Response options (and coding) were: always (4); often (3); sometimes (2); not often (1); never (0). Parents were also asked how often they or their spouse/partner participate in physical activity/sport together with their child (co-participation) [33]. Response options (and coding) were: never (0), less than once a week (0.5); once a week (1); 2–4 days a week (3); 5–6 days a week (5.5); every day (7). Parental modelling was assessed by asking parents how much time per week they participate in physical activities/sports in their leisure time 1) on weekdays; 2) on weekend days.[33] Response options (and coding) were: none at all (0); 30 min/wk (0.5); 1 hr/week (1); 2 hr/week (2); 3 hr/week (3); 4 hr/week (4); 5 hr/week or more (5). Responses were summed to compute hours/week of leisure-time physical activity. One-week test-retest reliability of all parent-reported items ranged from ICC = 0.72 to ICC = 0.88 in a separate sample of 316 parents.

#### Mediators

Children’s attitude to physical activity/sport (good/bad), perceived behavioural control (“I find doing physical activity/sports for one hour every day: very easy to very difficult”), health belief (“not exercising will make me fat”) and enjoyment (I like doing physical activity/sports”) of physical activity/sport were self-reported and measured with single items on five-point scales. These items have been described previously [33] and the one-week test-retest reliability of these items was acceptable [34]. Response options for each item were coded −2 to 2, with higher scores indicating a more positive attitude, stronger health belief and greater perceived behavioural control and enjoyment.

### Statistical analyses

Analyses were performed in 2011 using Stata/SE 11.1 (StataCorp LP, College Station, USA). Descriptive statistics (means, standard deviations and proportions) were computed to describe the sample and differences according to sex were examined using independent t-tests and chi-square analyses. Multi-level logistic regression was used to identify socio-demographic covariates. All subsequent analyses were adjusted for sex and significant covariates (child’s age, the responding parent’s education level). The analytical sample included only children with complete data for the dependent variable, sex and each family environment variable, mediator, and covariate (n = 4952). Compared to those excluded, a higher proportion of those in the analytic sample were girls (54% vs 49%, p < 0.001), participated in ≥ 30 min/week of sport (53% vs 48%, p < 0.001), had a dual parent family (93% vs 91%, p < 0.01), high level of parental education (responding parent: 60% vs 51%, p < 0.001) and were from Hungary (17% vs 7%), Norway (16% vs 10%) and Slovenia (18% vs 12%), compared to those who were excluded. A lower proportion were from Greece (14% vs 18%) or the Netherlands (7% vs 26%).

A series of multi-level logistic and linear regression analyses were performed using the xtmelogit and xtmixed commands to test for mediation by cognitive factors. Three-level nested models were specified (individual, school and country). From the logistic regression analyses, coefficients were used for the mediation analyses and odds ratios for descriptive purposes. Single mediation models were examined initially. First, associations between each family environment variable and sports participation were examined (c-path, xtmelogit). Second, associations between each family environment variable and each potential mediator were examined (a-path, xtmixed). Third, associations between each mediator and sports participation (b-path, xtmelogit) were examined, adjusted for the family environment variable (c’-path). The mediated effect of each mediator was computed using the product of coefficients method of multiplying coefficients for the a- and b-paths (a*b) [35]. Statistical significance (95% confidence intervals) of the mediated effect was determined using Sobel’s standard error (SE) formula (√ a^2 *^SE_b_^2^ + b^2^ * SE_a_^2^).

A multiple mediation model was constructed for each family environment variable by including all significant mediators in the single mediation models in the final regression model. Using coefficients from the b-paths of the multiple mediation model, individual mediated effects (a*b) were computed for each mediator and summed to compute the total mediated effect.[35] The percentage mediated was determined by dividing the total mediated effect by the sum of the direct effect (c’-path) and the total mediated effect (*Σ*[a_i_ * b_i_/(c ’ + *Σ*[a_i_ * b_i_)). The standard error was calculated using the delta method [36], using the equation, where COV stands for the covariance between the coefficients specified:

(1) $$\begin{array}{cc} SE_{\mathrm{\Sigma ai}*\mathrm{bi}} & =\surd \left(a_{1}^{2}*SE_{b1}^{2}+b_{1}^{2}+a_{2}^{2}*SE_{b2}^{2}+b_{2}^{2}*SE_{a2}^{2}\right)\\ & \left(+...+2*a_{1}*a_{2}*\mathrm{CO}V_{b1b2}+...\right) \end{array}$$

Percentage mediated was computed for each mediator in the multiple mediation model by dividing the individual mediated effect by the sum of the direct effect and total mediated effect a_i_ * b_i_/(c ’ + *Σ*[a_i_ * b_i_]).

## Results

### Sociodemographic characteristics

The sample comprised approximately equal proportions of boys and girls (Table 1). Most parent respondents were married or living with their partner, and had a high level of education. A majority of children had siblings living with them and spoke the native language of their country at home. There were few differences in socio-demographic factors between boys and girls.

**Table 1:** Sample characteristcs

	**Sex**		
	**Boys (n=2279)**	**Girls (n=2673)**	**Total sample (n=4952)**
Overall (%)^1^	46.0	54.0	100.0
Age (years; mean, sd)^2^	11.7 (0.7)	11.6 (0.7)*	11.6 (0.7)
Dual parental status (%)^1^	93.2	93.3	93.3
Married/living with partner (%)^1^	87.6	87.3	87.4
Siblings (%)^1^	83.0	83.1	83.0
Respondent’s highest level of education (%)^1^			
< 12 years	16.4	16.6	16.5
12–13 years	23.3	24.1	23.7
≥ 14 years	60.3	59.3	59.8
Native language most often spoken at home? ^1^	94.9	94.2	94.5
Country (%)^1^			
Belgium	12.9	13.7	13.4
Greece	13.5	14.2	13.9
Hungary	16.2	18.1	17.2
The Netherlands	7.2	6.7	7.0
Norway	16.0	15.5	15.8
Slovenia	18.6	17.9	18.2
Spain	15.6	13.8	14.6
**Sports participation**			
Participation ≥ 30mins/wk (%)^1^	61.7	46.4***	53.4
**Family environment (mean, sd)**^2^			
Number of PA equipment items [0–8]	5.2 (2.1)	5.5 (1.9)***	5.3 (2.0)
Financial support [0–4]	3.0 (1.3)	2.9 (1.4)***	2.9 (1.3)
Logistic support [0–4]	2.8 (1.3)	2.6 (1.4)***	2.7 (1.3)
Emotional support [0–4]	3.5 (0.9)	3.4 (1.0)***	3.4 (1.0)
Reinforcement	2.9 (1.1)	2.9 (1.1)	2.9 (1.1)
Parental modelling (hr/week) [0–10]	2.7 (2.6)	2.5 (2.5)*	2.6 (2.5)
Co-participation [0–7]	1.4 (1.3)	1.4 (1.4)	1.4 (1.4)
**Cognitions (mean, sd)**^2^			
Attitude [−2;2]	1.9 (0.4)	1.8 (0.4)**	1.9 (0.4)
Beliefs about weight gain [−2;2]	1.0 (1.3)	1.0 (1.3)	1.0 (1.3)
Enjoyment [−2;2]	1.8 (0.6)	1.7 (0.6)***	1.8 (0.6)
Perceived behavioural control [−2;2]	1.5 (0.8)	1.3 (0.9)***	1.4 (0.8)

*p ≤ 0.05; **p ≤ 0.01; ***p < 0.001.

^1^ Chi-square test of significance between boys and girls.

^2^ Independent sample t-tests between boys and girls.

Overall, 53% of participants participated in at least 30 min/week of sport, with a higher proportion of boys doing so compared to girls. In general, most family environment variables and mediators were more positive among boys than girls (Table 1).

### Family environment and sports participation (c-path)

As shown in Table 2, five of the seven family environment variables were positively associated with participation in ≥ 30 min/week of sport.

**Table 2:** Total and direct effects (OR, 95% confidence intervals) of family environment variables on duration of sports participation (>=30 mins/wk)

	**Total effect (c-path)**^**1**^	**Direct effect (c’-path)**^**2**^
	**OR (95% CI)**	**OR (95% CI)**
Number of PA equipment items	1.16 (1.12, 1.20)**	1.11 (1.08, 1.16)**
Financial support	1.53 (1.45, 1.61)**	1.44 (1.37, 1.52)**
Logistic support	1.47 (1.40, 1.55)**	1.40 (1.33, 1.47)**
Emotional support	1.51 (1.40, 1.62)**	1.39 (1.29, 1.50)**
Reinforcement	1.03 (0.98, 1.09)	1.01 (0.95, 1.07)
Parental modelling (hr/week)	1.07 (1.04, 1.10)**	1.06 (1.03, 1.08)**
Co-participation	1.01 (0.97, 1.06)	0.99 (0.94, 1.04)

* p < 0.01, ** p < 0.001: multi-level mixed effects linear regression (xtmelogit).

^1^ Model adjusted for covariates (child age, sex, responding parents’ highest education level).

^2^ Model adjusted for covariates and significant mediators (multiple mediator models).

### Mediation by cognitions

#### Family environment and potential mediators (a- path)

Each family environment variable was significantly positively associated with enjoyment of and perceived behavioural control for physical activity/sport (Table 3). Each family environment variable, except reinforcement and co-participation, was significantly positively associated with child attitude and beliefs about physical inactivity and becoming fat.

**Table 3:** **Results from single and multiple mediation models (B, 95% confidence interval) examining potential cognitive mediators of duration of sport participation (>=30 min/wk)**^**1**^

		**Single mediator models**		**Multiple mediator models**		
	**Path a B (95% CI)**	**Path b OR (95% CI)**	**Mediated effect (95% CI)**^**2**^	**Path b OR (95%CI)**	**Mediated effect (95% CI)**^**2**^	**Percent Mediated**^**3**^
**Physical activity equipment**						
Attitude	**0.018 (0.012, 0.023)**	**2.42 (2.05, 2.87)**	**0.024 (0.017, 0.031)**	**1.33 (1.10, 1.61)**	**0.008 (0.002, 0.013)**	6.6%
Beliefs about weight gain	**0.039 (0.019, 0.059)**	**1.11 (1.06, 1.16)**	**0.005 (0.002, 0.008)**	**1.07 (1.02, 1.12)**	**0.003 (0.001, 0.006)**	2.8%
Enjoyment	**0.020 (0.012, 0.028)**	**2.51 (2.20, 2.87)**	**0.031 (0.022, 0.040)**	**1.62 (1.39, 1.88)**	**0.016 (0.010, 0.023)**	12.8%
Perceived behavioural control	**0.034 (0.022, 0.046)**	**2.14 (1.97, 2.33)**	**0.036 (0.026, 0.047)**	**1.85 (1.69, 2.01)**	**0.029 (0.020, 0.038)**	21.0%
Total mediated effect					**0.056 (0.045, 0.067)**	33.9%
**Financial support**						
Attitude	**0.042 (0.033, 0.051)**	**2.27 (1.91, 2.69)**	**0.034 (0.024, 0.044)**	**1.30 (1.07, 1.58)**	**0.011 (0.003, 0.020)**	2.9%
Beliefs about weight gain	**0.041 (0.013, 0.069)**	**1.11 (1.06, 1.17)**	**0.004 (0.001, 0.008)**	**1.07 (1.02, 1.13)**	**0.003 (0.000, 0.006)**	0.8%
Enjoyment	**0.079 (0.067, 0.092)**	**2.29 (2.00, 2.61)**	**0.066 (0.051, 0.080)**	**1.50 (1.30, 1.74)**	**0.032 (0.020, 0.045)**	8.1%
Perceived behavioural control	**0.087 (0.069, 0.105)**	**2.08 (1.91, 2.26)**	**0.064 (0.049, 0.079)**	**1.83 (1.67, 2.00)**	**0.053 (0.039, 0.066)**	12.5%
Total mediated effect					**0.099 (0.082, 0.116)**	21.2%
**Logistic support**						
Attitude	**0.036 (0.027, 0.045)**	**2.35 (1.98, 2.79)**	**0.031 (0.021, 0.041)**	**1.34 (1.11, 1.63)**	**0.011 (0.003, 0.018)**	3.1%
Beliefs about weight gain	**0.032 (0.003, 0.060)**	**1.12 (1.07, 1.17)**	**0.004 (0.000, 0.007)**	**1.08 (1.03, 1.13)**	**0.002 (0.000, 0.005)**	0.7%
Enjoyment	**0.070 (0.057, 0.082)**	**2.37 (2.07, 2.71)**	**0.060 (0.046, 0.074)**	**1.53 (1.32, 1.78)**	**0.030 (0.018, 0.041)**	8.2%
Perceived behavioural control	**0.087 (0.070, 0.105)**	**2.09 (1.92, 2.27)**	**0.064 (0.049, 0.079)**	**1.82 (1.66, 1.99)**	**0.052 (0.039, 0.065)**	13.5%
Total mediated effect					**0.092 (0.076, 0.109)**	21.7%
**Emotional support**						
Attitude	**0.035 (0.023, 0.047)**	**2.43 (2.05, 2.87)**	**0.031 (0.019, 0.043)**	**1.35 (1.12, 1.63)**	**0.011 (0.003, 0.018)**	3.1
Beliefs about weight gain	**0.059 (0.021, 0.096)**	**1.11 (1.06, 1.17)**	**0.006 (0.001, 0.011)**	**1.07 (1.02, 1.12)**	**0.004 (0.000, 0.008)**	1.2
Enjoyment	**0.062 (0.046, 0.079)**	**2.47 (2.16, 2.82)**	**0.057 (0.039, 0.074)**	**1.59 (1.37, 1.85)**	**0.029 (0.017, 0.041)**	8.1
Perceived behavioural control	**0.088 (0.064, 0.112)**	**2.12 (1.95, 2.30)**	**0.066 (0.046, 0.085)**	**1.83 (1.67, 2.00)**	**0.053 (0.035, 0.070)**	13.9
Total mediated effect					**0.096 (0.076, 0.117)**	22.8%
**Reinforcement**						
Attitude	0.010 (-0.001, 0.020)	**2.57 (2.17, 3.04)**	0.009 (-0.001, 0.019)	-	-	-
Beliefs about weight gain	0.016 (-0.018, 0.050)	**1.12 (1.07, 1.18)**	0.002 (-0.002, 0.006)	-	-	-
Enjoyment	**0.016 (0.001, 0.031)**	**2.59 (2.27, 2.96)**	**0.015 (0.001, 0.030)**	**1.82 (1.58, 2.09)**	**0.009 (0.000, 0.019)**	50.3%
Perceived behavioural control	**0.025 (0.003, 0.047)**	**2.19 (2.01, 2.37)**	**0.020 (0.003, 0.037)**	**1.90 (1.74, 2.07)**	**0.016 (0.002, 0.030)**	63.1%
Total mediated effect					**0.026 (0.009, 0.042)**	73.1%
**Parental modelling (hr/week)**						
Attitude	**0.007 (0.002, 0.011)**	**2.55 (2.15, 3.01)**	**0.006 (0.002, 0.011)**	**1.37 (1.14, 1.66)**	**0.002 (0.000, 0.004)**	3.8%
Beliefs about weight gain	**0.030 (0.015, 0.045)**	**1.12 (1.07, 1.17)**	**0.003 (0.001, 0.005)**	**1.07 (1.02, 1.13)**	**0.002 (0.000, 0.004)**	3.8%
Enjoyment	**0.007 (0.001, 0.013)**	**2.58 (2.26, 2.95)**	**0.007 (0.001, 0.013)**	**1.63 (1.41, 1.89)**	**0.004 (0.000, 0.007)**	6.2%
Perceived behavioural control	**0.021 (0.012, 0.031)**	**2.17 (2.00, 2.36)**	**0.017 (0.009, 0.024)**	**1.85 (1.69, 2.02)**	**0.013 (0.007, 0.019)**	19.6%
Total mediated effect					**0.021 (0.014, 0.028)**	28.0%
**Co-participation**						
Attitude	0.006 (-0.002, 0.014)	**2.57 (2.18, 3.05)**	0.006 (-0.002, 0.014)	**-**	**-**	-
Beliefs about weight gain	0.010 (-0.016, 0.037)	**1.12 (1.07, 1.18)**	0.001 (-0.002, 0.004)	**-**	**-**	-
Enjoyment	**0.014 (0.002, 0.026)**	**2.60 (2.27, 2.96)**	**0.013 (0.002, 0.025)**	**1.82 (1.59, 2.09)**	**0.008 (0.001, 0.016)**	suppressed
Perceived behavioural control	**0.033 (0.016, 0.050)**	**2.19 (2.02, 2.38)**	**0.026 (0.012, 0.040)**	**1.90 (1.74, 2.07)**	**0.021 (0.010, 0.033)**	suppressed
Total mediated effect					**0.030 (0.016, 0.043)**	suppressed

Path a: association between family environment variable and mediator; Path b: association between mediator and sports participation.

^1^ Path a and path b adjusted for covariates (child age, sex, responding parents’ highest education level).

^2^ Mediated effect calculated from path b coefficient (not Odds Ratio).

^3^ Percentage mediated: a*b/c’+a*b.

**Bold:** significant associations.

#### Potential mediators and sports participation (b-path)

Attitude, beliefs about weight gain, physical activity/sport enjoyment and perceived behavioural control were positively associated with participation in ≥ 30 min/week of sport, independent of the family environment variables, in the single mediation models. All potential mediators included in multiple mediation models were also associated with sports participation, independent of the family environment variables.

#### Mediation effects (a*b)

In single mediation models, the cognitive factors mediated associations between each family environment variable and duration of sports participation, with few exceptions (attitude and beliefs did not mediate associations for reinforcement or co-participation) (Table 3). These latter mediators were excluded from the respective multiple mediation models. The total mediated effect was statistically significant in each of the multiple mediation models. The percentage of mediation explained was highest (73%) for reinforcement and ranged from 21% to 34% for the remainder of the variables. For co-participation, however, the model showed inconsistent mediation because the direct and indirect associations were opposite, possibly due to the small direct association. In general, perceived behavioural control contributed most to the mediated effect of each model, followed by enjoyment.

The five family environment variables with a significant total association also showed a significant direct association with sports participation. That is, the association remained significant after including all potential mediators (c’-path) in the multiple mediation models (Table 2), indicating partial mediation [36].

## Discussion

The findings confirm the importance of the family environment for children’s physical activity. Physical activity equipment items in the home, parental provision of financial, logistic and emotional support, and parental modelling were all positively associated with children’s participation in ≥30 min/week of sport. These associations were at least partly mediated by cognitions, as proposed in the EnRG framework [22]. This indicates that the influence of the family environment on children’s sport participation operates (at least in part) via children’s physical activity attitudes, beliefs, perceived behavioural control and enjoyment. The findings are consistent with other studies showing that cognitions mediate associations between elements of the family environment and physical activity in youth [21,27,28,30]. However, this study was the first to consider a range of cognitive mediators and sport participation in a large multi-national sample of European children.

The finding that the family environment was both directly and indirectly associated with children’s sports participation via different cognitions supports the dual process view outlined in the EnRG framework [22] that the environment influences behaviour through personal psychological constructs, and may also have an unmediated effect. For example, parental encouragement may have a direct influence on sports participation as it may prompt children to participate through a more automatic process without child deliberations regarding, for example, pros and cons or behavioural control beliefs. However, rather than indicating a degree of automaticity, it may be that direct effects were found because other unmeasured cognitive or personal factors are stronger mediators or also make a contribution to the combined mediated effect. It may also be that parents exert significant control of children’s behaviours at this age and that children have comparatively little autonomy.

For family environment variables with a significant total effect, the strongest associations were generally found for family (financial, logistic and emotional) support. This indicates that, for children, perhaps the most important aspect of the family environment to foster positive cognition toward sport is parental support, consistent with previous studies [25]. Further, repeated encouragement and other forms of support from parents may initiate and build confidence so that perceived behavioural control is enhanced or may make the positive consequences of sports more apparent so that attitude becomes more positive. The relatively low total proportion of behaviour mediated (< 34%) in this study, however, may be because children’s sport participation is less driven by cognitive processes than the behaviour of adolescents or adults. The establishment of positive cognitions towards sport participation during childhood may be particularly important for future participation as children gain autonomy and independence in choices about their leisure-time. It is also possible that the low proportion mediated could be due to measurement error given that the mediators were self-reported and were measured by single items rather than scales. Most likely, these family environment variables exert a mostly direct influence or can be explained by cognitive processes or innate preferences not measured in this study.

In general, the strongest mediated effect was found for perceived behavioural control. This is in contrast to the findings of van der Horst et al. [21], who found no evidence that perceived behavioural control mediates associations between the family environment and sports participation among adolescents. In this study, perceived behavioural control was measured by a single item asking how easy or difficult the child finds it to do physical activity/sport for an hour each day. It is perhaps not surprising that this item was the strongest mediator given that direct and indirect parental support and the provision of equipment provide favourable conditions that make it ‘easier’ for children to be active. Future studies should include control beliefs and specific forms of self-efficacy, such as barrier and instrumental self-efficacy.

Strengths of this study include the large sample of children from diverse countries across Europe and the inclusion of a wide range of family environment and cognitive variables. However, response rates differed between countries and there were several differences between the analytic sample and those excluded, which may have implications for generalizability. For example, the results may be less applicable to children whose parents have a low level of education. In addition, this study was cross-sectional and the findings are limited to sports participation rather than general physical activity. Our measure of sports participation may underestimate sports participation as only two ‘favourite’ sports could be reported and children’s understanding of the term ‘sport’ may exclude unorganised sports. However, this measure had good construct validity [34]. Despite this, correlational bias may have occurred as children self-reported each of the four mediators and their sports participation. Different, perhaps weaker, results may have been found if more objective measures of sports participation, such as parental report, were used. The study is further limited by the inclusion of only single-item measures of the family environment and the cognitive mediators.

## Conclusions

This study provides further impetus for the development of family-based interventions to increase children’s activity levels. These interventions should include strategies to change aspects of the family environment to be more supportive of children’s physical activity or sport, as this is likely to have a direct effect on sports participation, as well as foster enjoyment and other positive cognitions related to physical activity/sport. Future research should examine differences by country and how mediators change as children age and gain autonomy. Other aspects of the EnRG framework, such as components of automaticity and moderating effects of the family environment [22], could also be explored.

## Abbreviations

ENERGY: EuropeaN Energy balance Research to prevent excessive weight Gain among Youth; EnRG: Environmental Research framework for weight Gain prevention; Mins: Minutes; Sd: Standard deviation; SE: Standard error; TPB: Theory of Planned Behavior.

## Competing interests

The authors declare that they have no competing interests.

## Authors’ contributions

AFT conducted statistical analyses and drafted the manuscript. MMvS contributed to the development of measurement protocols and instruments, coordinated and supervised data collection in The Netherlands, assisted in data analysis and interpretation and critically revised the manuscript. JB conceived, designed and supervised the international study, contributed to the development of instruments, assisted in the interpretation of the analyses and critically revised drafts of the manuscript. EB contributed to the development of measurement protocols and instruments, coordinated and supervised data collection in Norway and critically revised the manuscript. MJMC contributed to the design of the international study and measurement protocols and instruments, coordinated and supervised data collection in the Netherlands, assisted in the interpretation of the analyses and critically revised the manuscript. IDB contributed to the design of the international study and the development of measurement protocols and instruments, coordinated and supervised data collection in Belgium and critically revised the manuscript. NJ contributed to the development of measurement protocols and instruments, coordinated and supervised data collection in Slovenia and critically revised the manuscript. LM contributed to the design of the international study and the development of measurement protocols and instruments, coordinated and supervised data collection in Belgium and critically revised the manuscript. YM contributed to the design of the study and to the development of measurement protocols and instruments, coordinated and supervised data collection in Greece and critically revised the manuscript. LAM contributed to the development of measurement protocols and instruments, coordinated and supervised data collection in Spain and critically revised the manuscript. JS contributed to the design of the study, assisted in the interpretation of the analyses and critically revised the manuscript. SJtV contributed to the conception and design of the international study and the development of measurement protocols and instruments, coordinated and supervised data collection in the Netherlands, assisted in data analysis and interpretation and critically revised the manuscript. All authors read and approved the final manuscript.
